# Impact of Maternal Hemoglobin Concentration on Fetal Outcomes in Adolescent Pregnant Women

**DOI:** 10.5812/ircmj.19670

**Published:** 2014-08-01

**Authors:** Leila Alizadeh, Azam Raoofi, Leili Salehi, Mani Ramzi

**Affiliations:** 1 Department of Maternal and Child Health, Ardabil Branch, Islamic Azad University, Ardabil, IR Iran; 2 Department of Health Education and Promotion, School of Public Health, Alborz University of Medical Sciences, Karaj, IR Iran; 3 Hematology Reaserch Center, Departement of Hematology and Medical Oncology, Shiraz University of Medical Sciences, Shiraz, IR Iran

**Keywords:** Hemoglobin, Pregnancy Outcome, Pregnancy, Adolescent, Apgar Score, Infant, Newborn

## Abstract

**Background:**

Studies on the association between maternal hemoglobin (Hb) concentration and adverse pregnancy outcome have been inconsistent. Many studies have shown the impact of Hb concentration on pregnancy outcomes in adult women; however, it is not revealed in adolescent pregnant women.

**Objectives:**

The aim of this study was to examine the effect of Hb concentration on birth outcomes in pregnant adolescents as a high-risk group.

**Patients and Methods:**

In this cross-sectional study, 312 healthy and nonsmoker adolescent pregnant women with gestational age (GA) of 37-40 weeks were chosen by random sampling, and were followed until delivery. A complete history was obtained from women. In addition, clinical examination and Hb test were performed. After birth, the associations between Hb concentration during pregnancy and birth outcomes were investigated. Statistical analyses were performed using SPSS software by t-test, chi-square and ANOVA.

**Results:**

In total, about 23.2 % were anemic, 58% had normal level of Hb (11-13.2 g/dL) and 18.8% had Hb > 13.2 g/dL. The mean birth weight was 3197.8 ± 398.25 grams and it was more in mothers with Hb = 10.5-12.5 g/dL than others (Hb < 10.5 or Hb > 12.5 g/dL) (P < 0.001). The lowest mean birth weight was found in mothers with Hb < 10.5 (3033.33 ± 422). Moreover, the mean birth weight of male newborns was more than females (P = 0.001). Eight percent of neonates had Apgar score less than eight. Low Apgar score in anemic group and mothers with high Hb concentration (Hb > 12.5 g/dL) was more than others.

**Conclusions:**

Abnormal Hb concentrations increase the risk of adverse birth outcomes such as low birth weight (LBW) and low Apgar scores in pregnant adolescents, so intensive care is recommended for this group of pregnant women.

## 1. Background

During pregnancy, increase in plasma volume exceeds the increase in red cell volume, which causes a physiological hemodilution, resulting in reduced Hb concentration (1). In normal pregnancy without iron supplementation, Hb concentration decreases from an average of 12.5–13.0 g/dL to an average of 11.0–11.5 g/dL (2). Anemia in pregnancy is a significant cause of direct and indirect morbidity and mortality, both for pregnant mother and her fetus (3). Therefore, iron supplementation is a primary component of pregnancy care. All supplementary programs in developing countries focus exclusively on iron supplementation (4). Studies on the association between maternal Hb level and adverse pregnancy outcomes have been inconsistent. Some studies showed a significant association between abnormal level of Hb concentration and adverse pregnancy outcomes including stillbirth, pregnancy induced hypertension, low birth weight (LBW), preterm delivery, and perinatal death (5-8), while some of them did not confirm such a correlation (9). Extreme maternal iron status may increase Hb level and adversely affect birth outcomes (4). However, some have doubted the necessity of iron supplementation and challenged its value (10, 11). Associations between Hb concentrations and birth outcomes have not been well characterized in adolescents despite the fact that this group is at a higher risk of early childbearing.

## 2. Objectives

The aim of this study was to examine birth outcomes in pregnant adolescents with different Hb concentrations. 

## 3. Patients and Methods

This cross-sectional study was conducted on pregnant women attending routine antenatal care at the largest primary health care clinic in Ardabil, a city located Northwest of Iran, during March 2012 to June 2013. In total, 312 subjects were selected from the center using systematic sampling method based on α = 0.05, S = 388 and d = 43. It is a governmental and referral center covering a large number of pregnant women from rural and urban areas of Ardabil. This study followed the ethical standards of the Helsinki declaration revised in 2008. The institutional (clinic) ethics committee approved the final study design. All subjects received necessary information about the study and an informed consent was obtained from them. Data gathering sheet did not contain the name of participants and the privacy of study was considered.

 Inclusion criteria were nonsmoker adolescent pregnant women under 18 years with gestational age (GA) of 37-40 weeks and singleton pregnancy. Exclusion criteria were smoking, having a disease associated with polycythemia such as asthma and chronic hypertension, fetal anomaly and a history of threatened abortion in the present pregnancy.

GA was calculated based on a reliable, self-reported estimate of the last menstrual period (LMP) or in the case of unknown LMP, an ultrasound performed early in pregnancy. Where both data were available with less than 14 days difference, we used LMP to estimate GA. Where the difference in two estimates exceeded 14 days, we considered ultrasound to estimate GA (1). Finally, two milliliters blood sample was drawn from each pregnant woman to evaluate Hb concentration. Laboratory criteria were used to measure the level of Hb concentration. Anemia was defined using the CDC (control of disease center) criteria for anemia during pregnancy (12). Based on these criteria, Hb cut-off value used to define anemia during the third trimester was 11.0 g/L.

Immediately after birth, Apgar score and birth weight were determined. Apgar score was estimated based on heart rate, muscular tone, nasal catheter response, and baby appearance (11). Birth weight was measured with 10 grams sensitivity. During the study, 36 mothers were excluded because of different reasons such as fetal death, preeclampsia, fetal anomaly and vaginal bleeding.

 Statistical analysis was performed using SPSS-16 package. Statistical significance was ascertained using χ2-test, t-test, and ANOVA. Besides, LSD post hoc test was used to assess differences between paired groups. All data had normal distribution. P < 0.05 was considered statistically significant. We used One-way ANOVA to find the association between Hb concentration and neonatal birth weight, because the dependent variable (weight) was quantitative with normal distribution in each group (P1 = 0.789, P2 = 0.280, P3 = 0.409), and three groups were independent. Homogeneity of variances was also checked while running the test (P = 0.175).

## 4. Results

In total, 312 healthy pregnant adolescent women with 37 < GA ≤ 40 were included in this study, among which 36 subjects were excluded and 276 cases participated until the final phase of the study (Table 1). The mean age of pregnant women was 16.37 ± 1.19 years and 92.3% of them were nulliparous. The prevalence of anemia was about 23.2% and most of them had mild anemia (9-10.9 g/dL). About 58% of women had a normal level of Hb and 18.8% had Hb more than 13.2 mg/dL. The mean birth weight of neonates was 3197.81 ± 398.25 grams and there was a significant correlation between birth weight and Hb concentration during the third trimester (Table 2). The mean birth weight in mothers with Hb = 10.5-12.5 g/dL was more than others (Hb < 10.5 g/dL or Hb > 12.5 g/dL) (P < 0.01). LSD post hoc results revealed a significant difference between neonatal mean birth weights of mothers whose Hb concentration was below 10.5 and those with 10.5-12.5 (P = 0.015). Nevertheless, there was no significant association between other groups. Moreover, the mean birth weight of male newborns was more than females (P = 0.001) (Table 3). There was a significant association between birth weight and GA (P = 0.001); however, we did not find any significant association between birth weight and maternal age (P = 0.5) or parity (P = 0.6). Eight percent of neonates had Apgar score less than eight and low Apgar score was significant in anemic mothers and those with high Hb concentrations (Hb ≥ 12.5 g/dL) (Table 2). Comparing pairs of groups only showed a significant difference between Apgar score of neonates of anemic mothers (Hb < 10.5) with mothers with high Hb concentrations (Hb ≥ 12.5 g/dL) (P = 0.003). We did not find any correlation between Apgar score with maternal age, parity, and fetal gender; however, there was a significant correlation between GA and Apgar score (P = 0.02). Apgar score less than eight in neonates born before 40 weeks of gestation was more than others (Table 3).

**Table 1.** Baseline Characteristics of Pregnant Adolescents and Their Newborns (n = 276) ^a^

Variables	Patients
**Maternal age, y**	
≤ 16	138 (50)
16-17.9	138 (50)
**Parity**	
Nulliparous	254 (92.03)
Multiparous	22 (7.97)
**Gestational age, weeks**	
37-40	191 (69.2)
≥ 40	85 (30.8)
**Hb concentration, g/dL**	
< 10.5	64 (23.19)
10.5-12.5	160 (57.97)
≥ 12.5	52 (18.84)
**Birth weight, g**	
< 2500	12 (4.35)
≥ 2500	264 (95.65)
**Apgar score**	
< 8	26 (9.42)
8-10	250 (90.58)
**Fetal gender**	
Male	140 (50.72)
Female	136 (49.28)

^a^ Data are presented as No. (%).

**Table 2.** Correlation Between Birth Outcomes and Maternal Hemoglobin Concentration ^a^

	Birth Weight, g		Apgar Score ≤ 8	
	Results	P Value ^b^	Results	P Value ^c^
**Hb concentration, g/dL**		0.03		0.02
< 10.5	3032.00 ± 420		7 (28)	
10.5-12.5	3239.62 ± 391		16 (10.1)	
≥ 12.5	3171.52 ± 394		4 (4.3)	

^a^ Data are presented as Mean ± SD or No. (%).

^b^ ANOVA.

^c^ Chi-square.

**Table 3.** Correlation Between Birth Outcome With Maternal and Neonatal Factors ^a^

Variables	Birth Weight			Apgar Score ≤ 8	
	Results	t Value	P Value ^b^	Results	P Value ^c^
**Maternal age, y**		0.6	0.81		0.158
< 16	3182 ± 394			9 (14.5)	
16-17.9	3212 ± 403			18 (8.5)	
**Parity**		0.4	0.644		0.072
Nulliparous	3197 ± 407			23 (8.9)	
Multiparous	3241 ± 289			4 (23.5)	
**Gestational age, w**		3.2	0.001		0.04
37-40	3150 ± 393			21 (12.7)	
≥ 40	3116 ± 385			6 (5.5)	
**Fetal Gender**		4.61	< 0.001		0.918
Male	3302 ± 368			14 (10)	
Female	3085 ± 402			13 (9.6)	

^a^ Data are presented as Mean ± SD or No. (%).

^b^ t-test.

^c^ Chi-square.

## 5. Discussion

Our study revealed a higher prevalence of anemia during pregnancy in adolescents; also they were at increased risk of adverse birth outcomes. Increased prevalence of anemia in this group occurred despite the fact that they received an average of nine prenatal visits, and prenatal supplements and additional iron supplementation were prescribed to all of them. Iron deficiency anemia is a major worldwide nutritional problem affecting all sections of population throughout the world (13). It is estimated that 50% of pregnant women in developing countries, 18% in industrialized countries, and up to 80% in South Asia have iron deficiency anemia (8). Moreover, previous studies found that teenagers had a significant higher risk of anemia (7, 14). Some studies found the effect of some factors such as maternal age, smoking, race, parity, and pregnancy intervals on Hb concentration during pregnancy (2, 15-17); therefore, subjects were selected from nonsmoker adolescents.

We found that the mean birth weight was less in anemic mothers (Hb < 10.5 g/dL) than mothers with normal Hb levels. Levy et al. studied a large population of 153396 pregnancies and reported that low Hb concentration during pregnancy (Hb < 10 g/dL) was associated with LBW and other adverse outcomes such as placental abruption, placenta previa, labor induction, previous cesarean section and non-vertex presentation (5). Another study reported that preconception Hb concentration affects pregnancy outcomes and the risk of LBW increased in mothers with anemia (18). Furthermore, a systematic review by Sukrat et al. revealed that Hb less than 11 g/dL increases the risk of preterm birth, LBW, and small for gestational age (SGA) (19).

Moreover, in our population, the mean birth weight was less in women with high Hb concentrations (Hb > 12.5 g/dL) than women with normal level of Hb. This is supported by two previous studies (7, 20). High Hb concentrations, due to inadequate plasma volume expansion, may increase blood viscosity, which leads to poor placental blood flow and compromised nutrient delivery to fetus, thus limiting the fetal growth (4). Besides, another study by Gonzales et al. revealed an association between high Hb concentration and adverse fetal outcomes such as stillbirth, preterm labor, and SGA in women who live both in low and high altitudes (6). A study by Hanieh et al. showed that inferior pregnancy outcomes are associated with higher Hb concentration in the third trimester (21).

Our data also indicated that male newborns tend to weight more than females. This result was supported by Restrepo et al. study without any known reason (22). In this study, we found a significant association between Hb concentration and birth Apgar scores. Apgar score less than eight increased in women with anemia. This supports the fact that poor placental and neonatal development in anemia is due to inadequate oxygen supply to fetus across the placenta as reported by other researchers (23).

Although many of our findings were in accordance with those reported for adult pregnant women, adolescents pregnant women had higher incidence of anemia compared to adults. On the other hand, high Hb concentrations not explained by smoking or preeclampsia were associated with increased risk of low infant birth weight and low Apgar score, like anemia. 

Our findings suggest that anemia, as a strong risk factor of negative pregnancy outcomes, should be considered and treated by practitioners to improve general public health. WHO recently recommended weekly iron–folic acid (IFA) supplementation to prevent anemia and adverse pregnancy outcomes (24, 25). These finding were also supported by a Cochrane systematic review showing positive pregnancy outcomes with intermittent IFA regimens (26). Our study provides the evidence that testing Hb concentration during pregnancy could prevent iron deficiency anemia during pregnancy, which would lead to positive pregnancy outcomes. Besides, due to high prevalence of anemia in adolescents and its important impact on public health, our study suggests practitioners to monitor adolescents for abnormal Hb concentrations during pregnancy to minimize the risk of adverse birth outcomes.

Our study had some strengths and limitations. There were some studies investigating the correlation of maternal anemia and fetal outcomes; however, to our knowledge, none of these studies investigated this association in adolescents. Besides, none of the previous studies assessed the association between Apgar score, as one of the important pregnancy outcomes, and Hb concentration during pregnancy, but it was investigated in ours. A limitation of this study was that we did not compare pregnancy outcomes in adolescent with adults. Future studies should focus on actual needs of iron supplementation in pregnant adolescents. 

## References

[1] Bodnar LM, Siega-Riz AM, Arab L, Chantala K, McDonald T (2004). Predictors of pregnancy and postpartum haemoglobin concentrations in low-income women.. Public Health Nutr..

[2] Rasmussen S, Bergsjo P, Jacobsen G, Haram K, Bakketeig LS (2005). Haemoglobin and serum ferritin in pregnancy--correlation with smoking and body mass index.. Eur J Obstet Gynecol Reprod Biol..

[3] Fraser D, Cooper MA, Myles MF (2009). Myles Textbook for Midwives..

[4] Ziaei S, Mehrnia M, Faghihzadeh S (2008). Iron status markers in nonanemic pregnant women with and without iron supplementation.. Int J Gynaecol Obstet..

[5] Levy A, Fraser D, Katz M, Mazor M, Sheiner E (2005). Maternal anemia during pregnancy is an independent risk factor for low birthweight and preterm delivery.. Eur J Obstet Gynecol Reprod Biol..

[6] Gonzales GF, Steenland K, Tapia V (2009). Maternal hemoglobin level and fetal outcome at low and high altitudes.. Am J Physiol Regul Integr Comp Physiol..

[7] Chang SC, O'Brien KO, Nathanson MS, Mancini J, Witter FR (2003). Hemoglobin concentrations influence birth outcomes in pregnant African-American adolescents.. J Nutr..

[8] Shobeiri F, Begum K, Nazari M (2006). A prospective study of maternal hemoglobin status of Indian women during pregnancy and pregnancy outcome.. Nutr Res..

[9] Ren A, Wang J, Ye RW, Li S, Liu JM, Li Z (2007). Low first-trimester hemoglobin and low birth weight, preterm birth and small for gestational age newborns.. Int J Gynaecol Obstet..

[10] Ziaei S, Janghorban R, Shariatdoust S, Faghihzadeh S (2008). The effects of iron supplementation on serum copper and zinc levels in pregnant women with high-normal hemoglobin.. Int J Gynaecol Obstet..

[11] Macedo A, Cardoso S (2010). [Routine iron supplementation in pregnancy].. Acta Med Port..

[12] (2007). Anemia Cutoffs during Pregnancy. CDC Prevention Guidelines Database..

[13] Patra S, Pasrija S, Trivedi SS, Puri M (2005). Maternal and perinatal outcome in patients with severe anemia in pregnancy.. Int J Gynaecol Obstet..

[14] Goonewardene IM, Deeyagaha Waduge RP (2005). Adverse effects of teenage pregnancy.. Ceylon Med J..

[15] Adebisi OY, Strayhorn G (2005). Anemia in pregnancy and race in the United States: blacks at risk.. Fam Med..

[16] Madhavan Nair K, Bhaskaram P, Balakrishna N, Ravinder P, Sesikeran B (2004). Response of hemoglobin, serum ferritin, and serum transferrin receptor during iron supplementation in pregnancy: a prospective study.. Nutrition..

[17] Dairo MD, Lawoyin TO (2004). Socio-demographic determinants of anaemia in pregnancy at primary care level: a study in urban and rural Oyo State, Nigeria.. Afr J Med Med Sci..

[18] Ronnenberg AG, Wood RJ, Wang X, Xing H, Chen C, Chen D (2004). Preconception hemoglobin and ferritin concentrations are associated with pregnancy outcome in a prospective cohort of Chinese women.. J Nutr..

[19] Sukrat B, Wilasrusmee C, Siribumrungwong B, McEvoy M, Okascharoen C, Attia J (2013). Hemoglobin concentration and pregnancy outcomes: a systematic review and meta-analysis.. Biomed Res Int..

[20] Scanlon KS, Yip R, Schieve LA, Cogswell ME (2000). High and low hemoglobin levels during pregnancy: differential risks for preterm birth and small for gestational age.. Obstet Gynecol..

[21] Hanieh S, Ha TT, Simpson JA, Casey GJ, Khuong NC, Thoang DD (2013). The effect of intermittent antenatal iron supplementation on maternal and infant outcomes in rural Viet Nam: a cluster randomised trial.. PLoS Med..

[22] Restrepo-Mesa SL, Estrada-Restrepo A, Gonzalez-Zapata LI, Agudelo-Suarez AA, Ronda-Perez E (2010). [Factors related to birth weight: a comparison of related factors between newborns of Spanish and Colombian immigrant women in Spain].. Arch Latinoam Nutr..

[23] Malhotra M, Sharma JB, Batra S, Sharma S, Murthy NS, Arora R (2002). Maternal and perinatal outcome in varying degrees of anemia.. Int J Gynecol Obstet..

[24] (2014). Intermittent iron and folic acid supplementation in non-anaemic pregnant women..

[25] (2012). Intermittent iron and folic acid supplementation in non-anaemic pregnant women..

[26] Pena-Rosas JP, De-Regil LM, Dowswell T, Viteri FE (2012). Intermittent oral iron supplementation during pregnancy.. Cochrane Database Syst Rev..

